# The Evil Effect upon Women of Prolonged Standing

**Published:** 1899-11

**Authors:** 


### *EDITORIAL NOTES AND COMMENTS.*

---

The Business office of THE JOURNAL-RECORD is 305, 306 Fitten Building.

The Editorial office is 318-319-320 The Prudential.

Address all Business communications to Dr. M. B. Hutchins, Mgr.

Make remittances payable to THE ATLANTA JOURNAL-RECORD OF MEDICINE.

On matters pertaining to the Editorial and Original Communications address Dr. Bernard Wolff, Atlanta.

Reprints of original articles will be furnished at cost price. Requests for the same should always be made *on the manuscript.*

We will present, post-paid, on request, to each contributor of an original article, twenty (20) marked copies of THE JOURNAL-RECORD containing such article.

---

### THE EVIL EFFECT UPON WOMEN OF PROLONGED STANDING.

AT the last meeting of the British Medical Association the following resolution was unanimously passed by that distinguished body: Resolved, "That this section recognizes the ill effects of long standing upon the health of women, and urges the Council to promote the passing of the bill as to providing seats for shop-girls (Scottish Section)."

This subject has, within the last two years, received considerable agitation at the hands of medical men and others in Endgland, and the importance of the same cannot but be justly recognized by all physicians. A bill for providing shop-girls with seats at their place of work has passed the House of Lords, which we understand from the *British Medical Journal* is tantamount to its becoming a working law. This bill was passed largely through the agitation promulgated by the medical men, who showed conclusively the ill effects upon women of standing long hours upon their feet in the various shops in which they were employed. This evil exists to a large extent in the United States, and more especially in the large cities, where a greater number of women are employed in retail stores. There is not a man or woman who, upon going into a large store where women are salesmen, has not been struck with the utterly tired and wan expression upon these poor, standing creatures. Not only do their appearances appeal thus, but medical statistics will show that such long standing is productive of ill health and disease.

Humanity itself appeals for seats for these poor working girls and women, that they may rest their tired frames during the long, weary working hours, amid an atmosphere which, in itself, is far from wholesome. Seats are provided for customers, who probably are far better off for an occasional standing upon their feet, while just across the counter the saleslady must stand the entire day. It is a barbarous custom, and the proprietors of stores should be forced by a humane public to allow and provide for seats for these working women. That such a condition of affairs is not purely theoretical, is proven by a paper recently read on this subject before the British Medical Association by Dr. J. Stuart Nairne, Surgeon to the Glasgow Samaritan Hospital for Women. He made a statistical study of 100 women who applied for treatment, and from this study he drew the following conclusions:

(a) " Seven per cent. of unmarried shop-girls only come for medical attendance, but

(b) " Forty per cent. of married women who have been shop-girls come under medical attention at a very early period in their married life, namely, mostly under 30 years of age.

(c) " Out of 7 per cent. of single women, 3 come with diseases especial to women, and out of 40 per cent. of married, 27 come with (d) same class of disease, so that we have a clear 30 per cent. of women who are or who have been shop-girls suffering from these special diseases.

(e) "Why should there be comparatively such a small percentage of single girls in active employment under treatment, and such a large percentage of married women? This looks very extraordinary at first sight, and seems to somewhat minimize the importance of the position I have maintained. A moment's consideration will, however, put this right. The girls in work are, as a rule, kept busy from morn till night; they are young, and have not as yet exhausted their energies; they have little time to rest and no time to complain. Parents and masters are alike frequently unobservant of the early symptoms of exhaustion and overwork. The girls themselves are anxious to work; they like to be considered strong; the world is before them, and much lighter if they look or pretend to be well and healthy. They are much more likely to form a suitable 'match' if they are strong, and therefore they go on, and only the small 3 per cent. come for advice and assistance who can go on no longer.

(f) "Now with regard to the 40 per cent., the alarmingly large proportion of 27 who suffer from special uterine and ovarian disease, shows emphatically that it is no chance illness, but that it must be a regular and frequent consequence of some condition of their daily work. One of the conditions—and I have no hesitancy in saying it—one of the most potent is the long period of standing and hanging about on their feet.

"Statistics may proverbially be made to prove anything, but in this case I am only dealing with facts, and supplying the proportionate numbers of these facts. I have indicated a definite number of shop-girls, having no way specially selected them, and I have shown that as a statistical fact they are burdened with a definite number of special diseases. With the short time at my disposal it is impossible to deal with a larger number of cases, but if it were necessary I would do it; it is not necessary. I am quite aware that a thousand would give identical results. The girls are broken down and wearied while in active work. They keep at their work by force of circumstances. They get married and presumably have an easier time of it; but their former hard conditions now tell on them. They suffer from inflammation and displacements and dislocations, and the new order of things, instead of mending matters, makes them worse, and they can endure no longer."

Such facts as these must evidently have weight, and show the injurious influence of long standing hours. ROY.

